# Identification of a six-lncRNA signature associated with recurrence of ovarian cancer

**DOI:** 10.1038/s41598-017-00763-y

**Published:** 2017-04-07

**Authors:** Kai Yang, Yan Hou, Ang Li, Zhenzi Li, Wenjie Wang, Hongyu Xie, Zhiwei Rong, Ge Lou, Kang Li

**Affiliations:** 1 Department of Epidemiology and Biostatistics, School of Public Health, Harbin Medical University, Harbin, 150086 P.R. China; 2 Key Laboratory of Cardiovascular Medicine Research, Harbin Medical University, Ministry of Education, Harbin, 150086 P.R. China; 3 Department of Gynecology Oncology, the Tumor Hospital, Harbin Medical University, Harbin, 150086 P.R. China

## Abstract

Ovarian cancer (OvCa) is the leading cause of death among all gynecological malignancies, and recurrent OvCa is almost always incurable. In this study, we developed a signature based on long non-coding RNAs (lncRNAs) associated with OvCa recurrence to facilitate personalized OvCa therapy. lncRNA expression data were extracted from GSE9891 and GSE30161. LASSO (least absolute shrinkage and selection operator) penalized regression was used to identify an lncRNA-based signature using the GSE9891 training cohort. The signature was then validated in GSE9891 internal and GSE30161 external validation cohorts. The Database for Annotation, Visualization and Integrated Discovery (DAVID) was used to explore the possible functions of identified lncRNAs. A six-lncRNA signature (RUNX1-IT1, MALAT1, H19, HOTAIRM1, LOC100190986 and AL132709.8) was identified in the training cohort and validated in internal and external validation cohorts using the LASSO method (P < 0.05). This signature was also independent of other clinical factors according to multivariate and sub-group analyses. The identified lncRNAs are involved in cancer-related biological processes and pathways. We selected a highly reliable signature based on six lncRNAs associated with OvCa recurrence. This six-lncRNA signature is a promising method to personalize ovarian cancer therapy and may improve patient quality of life quality according to patients’ condition in the future.

## Introduction

Ovarian cancer (OvCa) is a common gynecological malignancy and the commonest cause of gynecological cancer-associated deaths in developed countries^1^. It is estimated that there will be 22,280 new cases and 14,240 deaths attributed to OvCa in the United States in 2016^2^. Although there is a high initial response rate to standard surgery and chemotherapy, most OvCa patients will develop recurrence within 18 months after standard first-line treatment^3^. More seriously, recurrent OvCa usually develops into platinum-resistant disease and is almost always incurable^4^. Therefore, stratification of patients to identify high-risk patients may provide more effective treatment strategies and personalized therapies.

Long non-coding RNA (lncRNA) is a class of non-coding RNAs that are longer than 200 nucleotides in length^5^. Increasing studies have showed that abnormal expression of lncRNAs is associated with human cancers and that some play important roles in a variety of biological processes in cancer. Currently, several lncRNA-based signatures have been identified as prediction of patient survival in several cancers, such as gastric cancer^6^, lung cancer^7^, breast cancer^8^, colorectal cancer^9^ and esophageal squamous cell cancer^10^. Recent studies also indicated that lncRNAs were associated with OvCa recurrence and survival^11^. For example, lncRNAs CCAT2, HOTAIR, AB073614, and ANRIL have been demonstrated to be associated with poor prognosis of OvCa^12–15^. A recent study identified an eight-lncRNA signature associated with overall survival (OS) of OvCa based on The Cancer Genome Atlas (TCGA)^16^.

In this study, we used LASSO (least absolute shrinkage and selection operator) penalized regression to identify a six-lncRNA signature associated with OvCa recurrence based on a training cohort. Then we validated it in internal and external validation cohorts, analyzed it in sub-groups of OvCa patients, and demonstrated this signature was independent from other clinical factors. We also analyzed the correlation between the signature and OS of OvCa patients. Furthermore, we found that these lncRNAs were involved in biological processes (e.g., cell adhesion, inflammatory response and immune response) and pathways (e.g., ECM-receptor interaction, focal adhesion and cell adhesion molecules) in cancers. Thus, our six-lncRNA signature may be a promising method to stratify OvCa patients and identify those at high-risk in the future.

## Results

### Demographic and clinical characteristics

The detailed demographic and clinical characteristics are listed in Supplementary Table S1. A total of 311 OvCa patients were included in our study, including 100 in the GSE9891 training cohort, 157 in the GSE9891 internal validation cohort and 54 in the GSE30161 external validation cohort. The median ages (ranges) of the three cohorts were 58 (23–80), 60 (33–80), and 62 (38–84) years, respectively. Seventy-four (74%), 111 (71%), and 48 (89%) patients relapsed during follow-up, respectively. The tumor stage, tumor grade and histology type are also summarized in Supplementary Table S1.

### Identification of lncRNA signature and generation of risk score

To identify lncRNAs associated with OvCa recurrence, LASSO penalized regression was performed using lncRNA expression data. This method can select an optimal subset of lncRNAs without collinearity by imposing a penalty and shrinking most regression coefficients to zero. After 100 times of 10-fold cross validation, the optimal tuning parameter lambda1 was 16.9841 in our study. As a result, the regression coefficients of six lncRNAs were not zero when lambda1 was 16.9841, and we selected these six lncRNAs as signatures associated with OvCa recurrence (Table 1). We also conducted univariate cox regression for the six lncRNAs, in which the regression coefficients were consistent with that in LASSO penalized regression, and all six lncRNAs were statistically significant (P < 0.05). Of these six lncRNAs, five were positively associated with DFS (high expression of these lncRNAs led to a high-risk score and shorter survival) and one was negatively associated with DFS (the high expression of this lncRNA led to a low-risk score and longer survival).

**Table 1:** Overall information of six prognostic lncRNAs associated with DFS in GSE9891 training cohort (n = 100).

Probe	Gene symbol	Chromosomal location	C^a^	C^b^	P^b^	HR^b^
220918_at	RUNX1-IT1	Chr21q22.12	0.1078	0.5589	<0.0001	1.749
223940_x_at	MALAT1	Chr11q13.1	0.0751	0.1514	0.0410	1.163
224646_x_at	H19	Chr11p15.5	0.1083	0.1625	0.0004	1.176
228642_at	HOTAIRM1	Chr7p15.2	0.1110	0.2776	0.0011	1.320
235167_at	LOC100190986	Chr16p12.2	−0.0155	−0.3217	0.0061	0.725
242856_at	AL132709.8	Chr14q32.31	0.0195	0.2975	0.0016	1.346

^a^Derived from the LASSO penalized regression in 100 patients of GSE9891 training cohort. ^b^Derived from the univariate Cox proportional hazards regression in 100 patients of GSE9891 training cohort.

Abbreviations: C Coefficient, P P value, HR Hazard Ratio.

**Figure 1:**
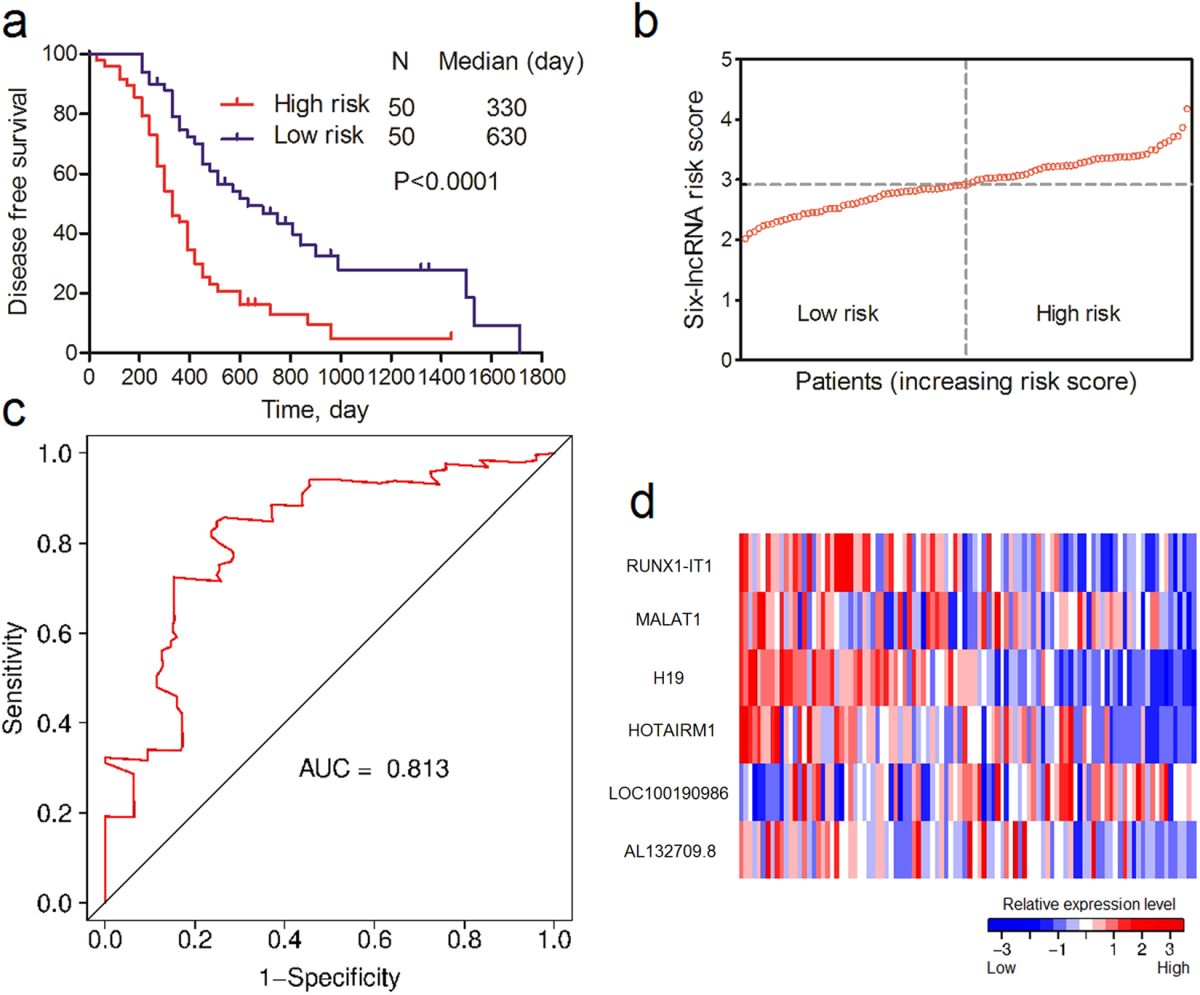
Association between the six-lncRNA signature and DFS of OvCa patients in GSE9891 training cohort. (**a**) K-M curve of DFS between low- and high-risk patients. (**b**) Risk scores of each patient in the GSE9891 training cohort (sorted by risk score). (**c**) Time-dependent ROC curve analysis of the DFS prediction based on the risk score with three years as the time point. (**d**) Expression heat map of the six lncRNAs in OvCa patients in the GSE9891 training cohort (sorted by risk score).

To identify low- and high-risk patients for OvCa recurrence, we developed a prognostic model based on the expression of six lncRNAs and their regression coefficient in LASSO penalized regression as follows: Risk score = (0.1078*RUNX1-IT1) + (0.0751*MALAT1) + (0.1083*H19) + (0.111*HOTAIRM1) − (0.0155*LOC100190986) + (0.0195*AL132709.8). The patients were then divided into low- and high-risk groups according to the median risk score value (2.9232). As a result, the patients in the low-risk group had a better survival outcome than the high-risk group, as shown in Fig. 1a (P < 0.0001). The area under the curve (AUC) of time-dependent ROC curves for the risk score was 0.813 at three years (Fig. 1c). These results demonstrated a good performance of our six-lncRNA signature in predicting DFS for OvCa patients. Risk scores and relative expression levels of all patients are shown in Fig. 1b,d.

### Validation of the six-lncRNA signature in the GSE9891 internal validation and entire cohorts

**Figure 2:**
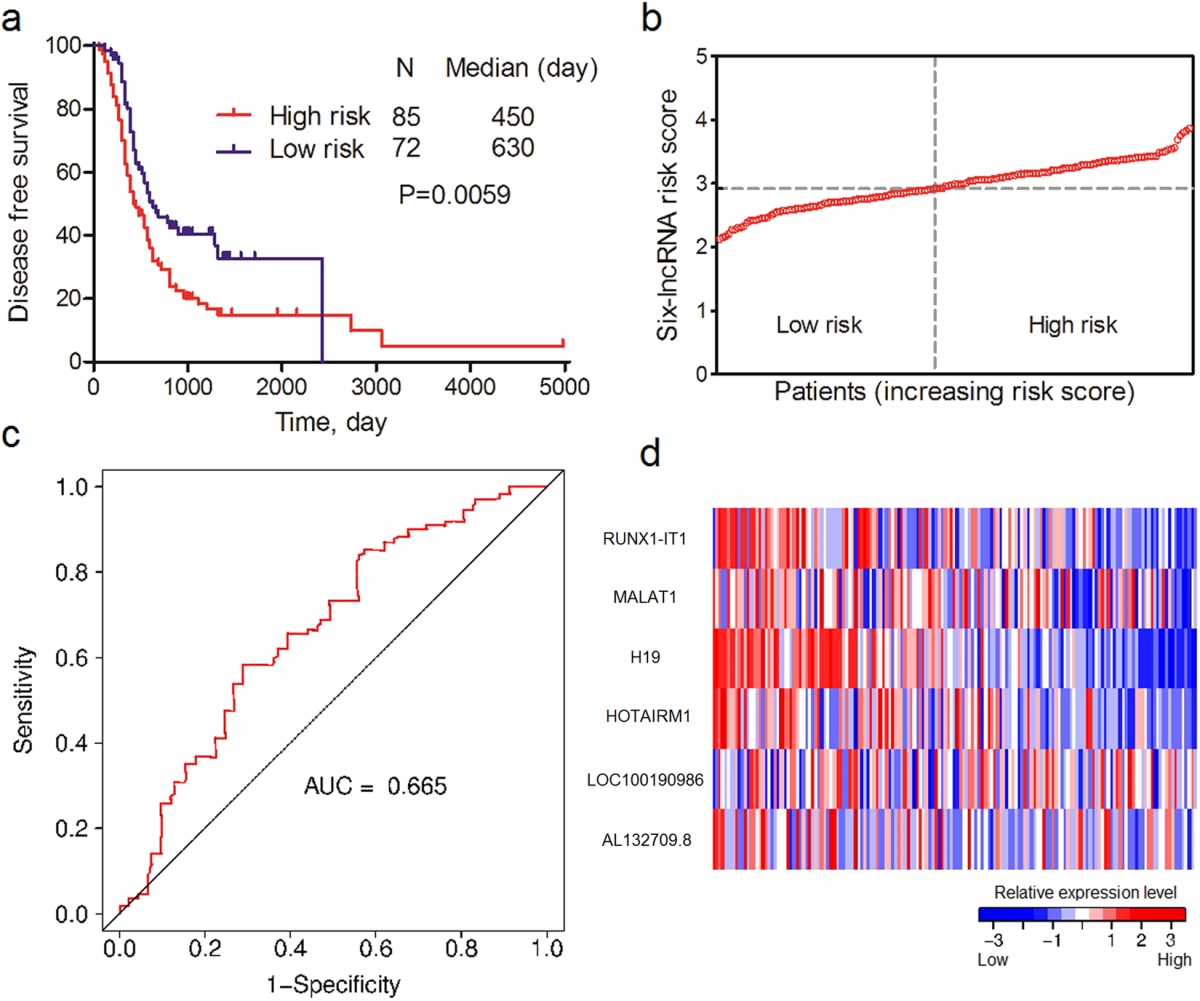
Association between the six-lncRNA signature and DFS of OvCa patients in GSE9891 internal validation cohort. (**a**) K-M curve of DFS between low- and high-risk patients. (**b**) Risk scores of each patient in the GSE9891 internal validation cohort (sorted by risk score). (**c**) Time-dependent ROC curve analysis of the DFS prediction based on the risk score with three years as the time point. (**d**) Expression heat map of six lncRNAs in OvCa patients in the GSE9891 internal validation cohort (sorted by risk score).

To confirm the ability of the six-lncRNA signature in predicting DFS for OvCa patients, we validated it in the GSE9891 internal validation and entire cohorts. The same risk formula and cutoff value were used to calculate risk scores and divide the patients into low- and high-risk groups. The results of the two cohorts were consistent with the GSE9891 training cohort. Patients with higher risk scores had poorer DFS. The differences of survival curves between the two groups were statistically significant in two cohorts (Fig. 2a and Supplementary Fig. S1a). The AUCs of the two cohorts were 0.665 and 0.697, respectively (Fig. 2c and Supplementary Fig. S1c). Risk scores and relative expression levels of all patients in the two cohorts are separately shown in Fig. 2b,d and Supplementary Fig. S1b,d.

### Further validation of the six-lncRNA signature in the GSE30161 external validation cohort

**Figure 3:**
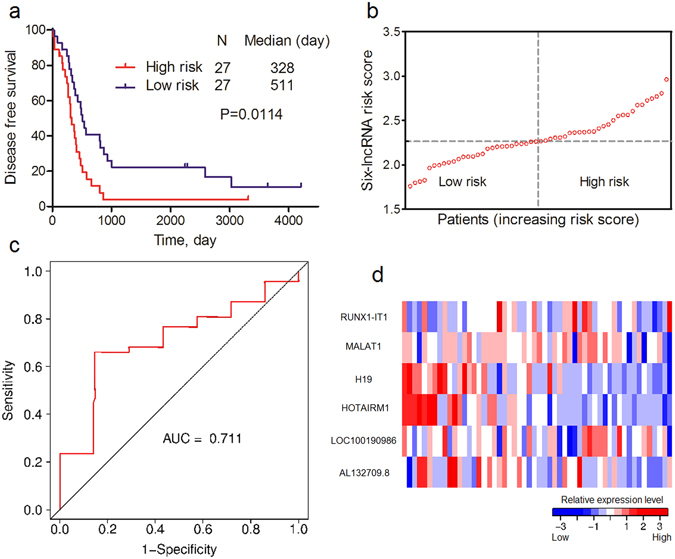
Association between six-lncRNA signature and DFS of OvCa patients in GSE30161 external validation cohort. (**a**) K-M curve of DFS between low- and high-risk patients. (**b**) Risk scores of each patient in GSE9891 internal validation cohort (sorted by risk score). (**c**) Time-dependent ROC curve analysis of the DFS prediction based on the risk score with three years as the time point. (**d**) Expression heat map of six lncRNAs in OvCa patients in the GSE30161 external validation cohort (sorted by risk score).

We further validated our findings in GSE30161 external validation cohort. The same risk formula was used to calculate risk scores for every OvCa patient. The median risk score value in GSE30161 (2.2672) was used to divide the patients into low- and high-risk scores. Similar to the result in GSE9891, the patients in low-risk group also had a better survival outcome than those in the high-risk group, as shown in Fig. 3a (P = 0.0114). The AUC of time-dependent ROC curves for the risk score was 0.711 at three years (Fig. 3c). Risk scores and relative expression levels of all patients are also shown in Fig. 3b,d.

### Sub-group analysis of six-lncRNA signature in GSE9891 and GSE30161

**Figure 4:**
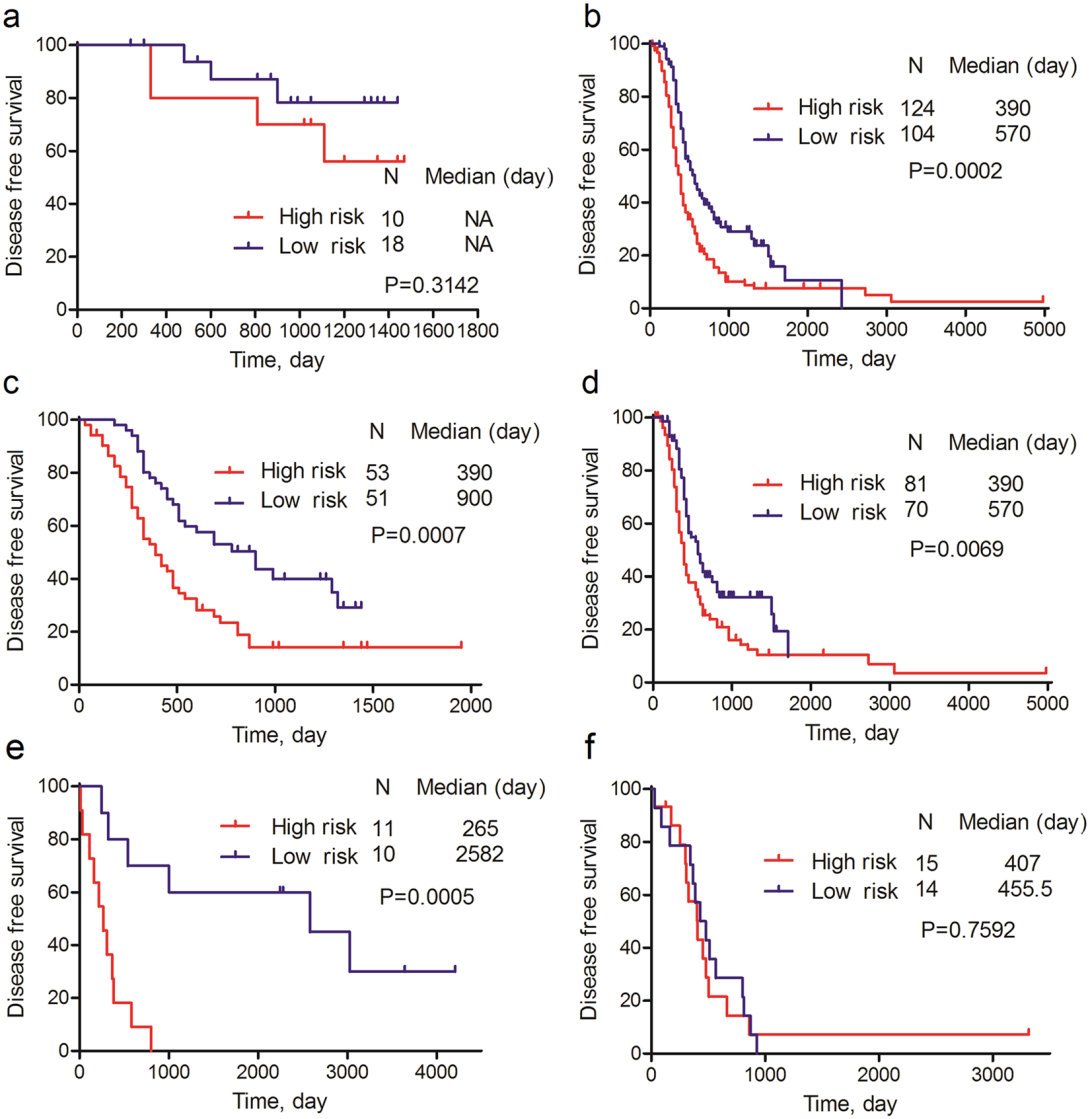
Sub-group analysis of association between six-lncRNA signature and DFS of OvCa patients. (**a**) K-M curve of early-stage OvCa patients in GSE9891. (**b**) K-M curve of late-stage OvCa patients in GSE9891. (**c**) K-M curve of low-grade OvCa patients in GSE9891. (**d**) K-M curve of high-grade OvCa patients in GSE9891. (**e**) K-M curve of low-grade OvCa patients in GSE30161. (**f**) K-M curve of high-grade OvCa patients in GSE30161.

We then analyzed the six-lncRNA signature in sub-groups of OvCa patients. As shown in Fig. 4, the differences of K-M survival curves between the low- and high-risk patients were statistically significant (P < 0.05) for late-stage (Fig. 4b), low-grade (Fig. 4c), high-grade (Fig. 4d) OvCa patients in the GSE9891 and low-grade (Fig. 4e) OvCa patients in the GSE30161. For the early-stage patients in GSE9891 (Fig. 4a) and high-grade patients in GSE30161 (Fig. 4f), the differences of K-M survival curves were not statistically significant, but patients in the low-risk group still tended to have a better DFS. The ROCs of these sub-groups were in Supplementary Fig. S2.

### Independence of six-lncRNA signature and other clinical factors

To assess whether the six-lncRNA signature was independent of other clinical factors, univariate and multivariate cox regression analyses were conducted in each patient cohort including risk scores for the six lncRNAs, age, tumor stage, tumor grade and histology type. In univariate cox regression, risk score and tumor stage were significantly associated with DFS. After adjusting for age, tumor stage, tumor grade and histology type, risk score still maintained a significant correlation with DFS in all GSE9891 and GSE30161 cohorts (Table 2).

**Table 2:** Univariate and multivariate cox regression analyses of DFS in GSE9891 and GSE30161.

	Univariate analysis				Multivariate analysis			
Variable	C	P	HR	95% CI of HR	C	P	HR	95% CI of HR
**GSE9891 training cohort (N = 100)**								
Risk score	1.667	**<0.0001**	5.296	2.925–9.589	1.6849	**<0.0001**	5.392	2.84–10.237
Age	−0.0062	0.6223	0.994	0.970–1.019	−0.0015	0.9129	0.999	0.973–1.025
Stage	1.7276	0.0170	5.627	1.362–23.251	1.4465	0.0554	4.248	0.967–18.656
Grade	−0.0052	0.9828	0.995	0.620–1.595	0.1633	0.5308	1.177	0.707–1.962
Histology subtype	0.5031	0.2811	1.654	0.663–4.128	−0.1193	0.8088	0.888	0.338–2.332
**GSE9891 internal validation cohort (N = 157)**								
Risk score	0.79	**0.0016**	2.203	1.351–3.594	0.5924	**0.0255**	1.808	1.075–3.041
Age	0.0183	0.0699	1.018	0.999–1.039	0.0152	0.1542	1.015	0.994–1.037
Stage	1.7095	0.0002	5.526	2.246–13.597	1.5578	0.0009	4.748	1.899–11.871
Grade	0.2027	0.3086	1.225	0.829–1.809	−0.1177	0.5633	0.889	0.596–1.325
Histology subtype	1.5048	0.0103	4.503	1.426–14.22	0.846	0.1606	2.33	0.715–7.597
**GSE9891 entire cohort (N = 257)**								
Risk score	1.0096	**<0.0001**	2.745	1.902–3.962	0.9028	**<0.0001**	2.466	1.689–3.601
Age	0.0087	0.2618	1.009	0.994–1.024	0.006	0.4587	1.006	0.99–1.022
Stage	1.7306	<0.0001	5.644	2.642–12.058	1.6227	<0.0001	5.067	2.337–10.985
Grade	0.1245	0.4151	1.133	0.839–1.528	−0.0524	0.7384	0.949	0.698–1.291
Histology subtype	0.9768	0.007	2.656	1.305–5.404	0.3133	0.3998	1.368	0.66–2.837
**GSE30161 entire cohort (N = 54)**								
Risk score	1.1528	**0.0309**	3.167	1.112–9.022	1.5104	**0.0114**	4.528	1.405–14.597
Age	0.0037	0.8029	1.004	0.975–1.034	0.0064	0.7046	1.006	0.974–1.041
Stage	—	—	—	—	—	—	—	—
Grade	0.4346	0.1765	1.544	0.822–2.9	0.7009	0.0429	2.015	1.023–3.972
Histology subtype	0.3131	0.4456	1.368	0.612–3.057	1.2788	0.0283	3.592	1.146–11.26

Abbreviations: C Coefficient, P P value, HR Hazard Ratio, CI Confidence Interval.

### Correlation between the six-lncRNA signature and OS

In addition to the analyses of association between the signature and OvCa recurrence, we also explored the correlation between the signature and OS. In accordance with the results of DFS, patients with higher risk scores had poorer OS. The differences of survival curves between the two groups were statistically significant in GSE9891 training, GSE9891 entire and GSE30161 external validation cohorts (Supplementary Fig. S3a,c,d). The differences of K-M survival curves were not statistically significant in GSE9891 internal validation cohort (P = 0.0612), but patients in the low-risk group tended to have a better OS (Supplementary Fig. S3b). After adjusting for age, tumor stage, tumor grade and histology subtype, risk score still maintained a significant correlation with OS in GSE9891 training and entire cohorts. The patients in the low-risk group in GSE9891 internal validation and GSE30161 external validation cohorts still tended to have a better OS (P < 0.08) (Supplementary Table S2).

### Functional characteristics of six lncRNAs

**Figure 5:**
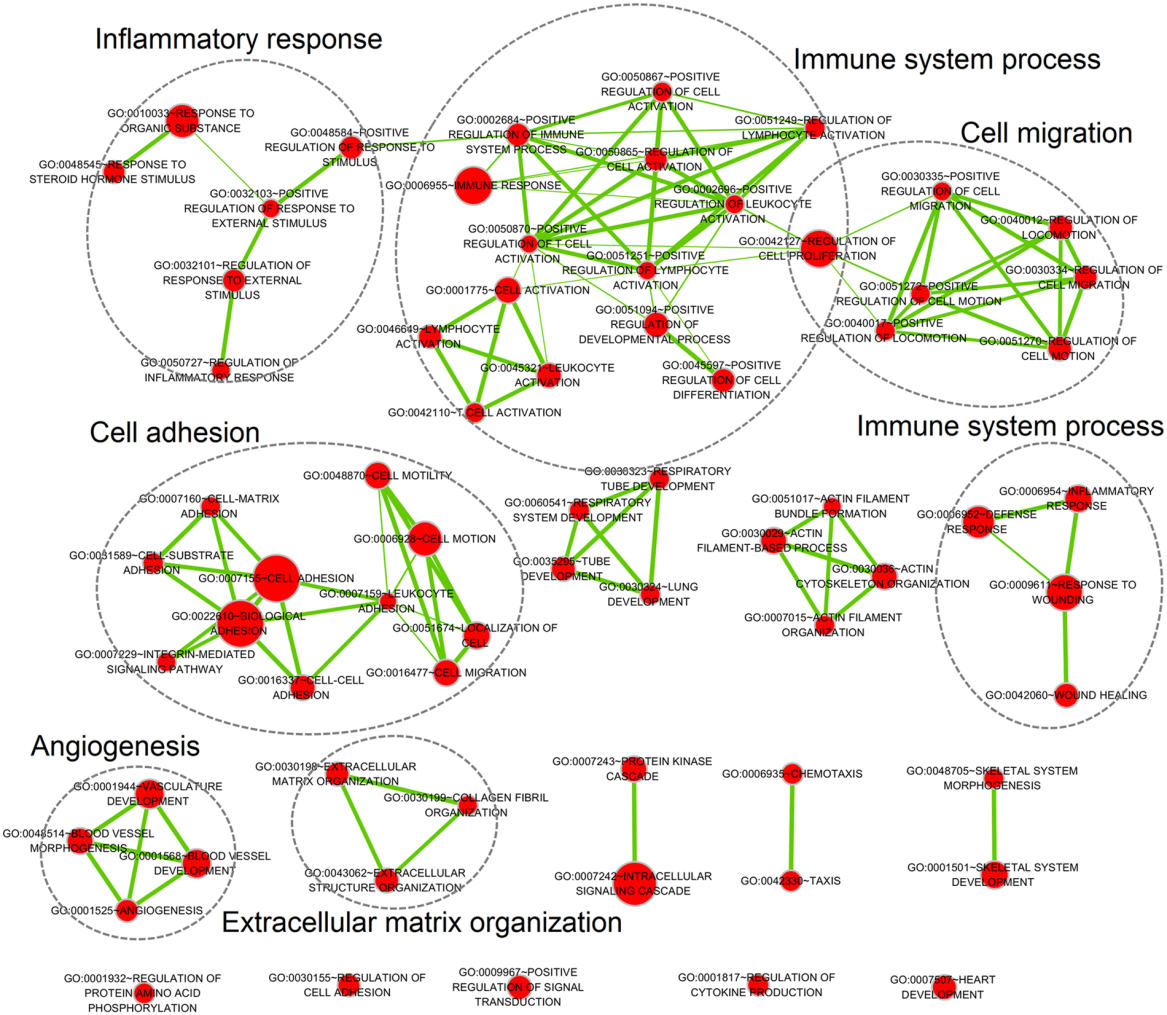
Interaction network of significant GO biological process terms with similar functions associated with six lncRNAs. Red nodes represent GO biological process terms. Node size is proportional to the total number of DEGs in that term. Thickness of green lines is proportional to the shared DEGs of two connected terms.

We further analyzed the possible functions associated with the six identified lncRNAs in OvCa by GO and KEGG functional enrichment analysis using the DAVID tool. Using Satterthwaite t-test and FDR correction, 3814 genes were found to be differentially expressed between the low- and high-risk groups. These DEGs were considered as genes associated with six lncRNAs. After functional annotation in DAVID, 68 GO biological process (BP) terms, 27 GO cellular component (CC) terms, 18 GO molecular function (MF) terms, and 6 KEGG pathways were significantly enriched with FDR < 0.05 (Supplementary Tables S3–S6). The enriched BP terms were involved in some important biological processes in cancer, such as blood vessel development, inflammatory response and immune response (Table 3). The enriched KEGG pathways were also involved in cancer-related pathways, such as ECM-receptor interaction, focal adhesion and cell adhesion molecules (Table 3). An interaction network of significant BP terms with similar function showed the six lncRNAs were mainly associated with inflammatory response, immune system process, cell migration, cell adhesion, angiogenesis and extracellular matrix organization (Fig. 5). Most of the enriched GO terms and KEGG pathways have been found to be closely associated with OvCa initiation and progression, which could increase the credibility of the six-lncRNA signature from a biological perspective.

**Table 3:** Top 10 significant GO BP terms and KEGG pathways enriched with DEGs in OvCa.

Rank	Biological Process	FDR	KEGG pathway	FDR
1	Cell adhesion	1.10E-24	ECM-receptor interaction	7.30E-10
2	Biological adhesion	1.40E-24	Focal adhesion	4.00E-09
3	Response to wounding	2.80E-15	Cell adhesion molecules (CAMs)	7.50E-04
4	Vasculature development	2.10E-13	Leukocyte transendothelial migration	2.60E-03
5	Blood vessel development	2.30E-12	Cytokine-cytokine receptor interaction	2.70E-03
6	Inflammatory response	5.10E-10	Chemokine signaling pathway	4.50E-02
7	Immune response	6.30E-10		
8	Extracellular matrix organization	1.40E-09		
9	Blood vessel morphogenesis	3.00E-09		
10	Regulation of response to external stimulus	3.30E-09

## Discussion

OvCa is the sixth commonest cause of female cancer death in developed countries^1^. Approximately 75% of women with OvCa present with late-stage disease, most (78.79% in this study) of whom will develop recurrence^4, 17^. Treatment of OvCa is based on a combination of surgery and chemotherapy, and standard treatment for late-stage OvCa combined platinum-based chemotherapy with cytoreductive surgery can achieve a good result^18^. However, for most late-stage patients, cancer recurrence seems to be inevitable. Moreover, recurrent OvCa is often insensitive to chemotherapy and is generally incurable^4^. Thus, it is of great importance to identify low- and high-risk patients to enable improved treatment. Once we can identify high-risk patients, we can adopt a more effective personalized treatment strategy, such as novel drugs and targeted therapies, to delay the recurrence of cancer and improve patient quality of life^18^.

In our study, LASSO penalized regression was used to identify six lncRNAs associated with OvCa recurrence. LASSO penalized regression is optimized to high-dimensional data, which can select a few of the most influential variables and avoid collinearity of variables at the same time. These properties are not available in many other univariate and multivariate methods. lncRNA may be superior biomarkers in cancer as it is a new area of molecular biology, does not encode proteins, and may have better specificity^19^. In addition to the inherent virtues of LASSO and lncRNAs, we also validated the six-lncRNA signature in internal and external cohorts, and certified its credibility from a biological perspective. The signature also showed a great ability to stratify OvCa patients into low- and high-risk subgroup with significantly different overall survival.

Further analysis, including sub-group analysis and adjustment of clinical factors, indicated that the six-lncRNA signature could still predict recurrence in most sub-groups and were independent of other clinical factors, including age, tumor stage, tumor grade and histology type. It is well known that the prognosis of late-stage OvCa patients is worse than early-stage OvCa patients (Fig. 4a,b). In our study, the six-lncRNA signature could identify all high-risk patients with late-stage OvCa in different cohorts (Figs 3a and 4b). This could be of great use to improve the prognosis of these severe cancer patients.

We also explored the possible functions of these genes. Although the exact mechanism of most of the six lncRNAs was unclear, the lncRNAs were still closely related with cancer, as described in the literature, which strengthened the reliability and possibility of our six-lncRNA signature as a predictor of OvCa recurrence. In addition to the reports in the literature, our functional enrichment analysis of the six lncRNA-related genes may also shed new light on the possible functions of these lncRNAs in OvCa.

RUNX1-IT1, MALAT1, H19, and HOTAIRM1 have been widely associated with cancer in recent years. RUNX1-IT1 is an oncogenic lncRNA that can promote tumor progression and metastasis^20^. RUNX1-IT1 was overexpressed in non-responder chronic myeloid leukemia^21^. MALAT1 was found to be upregulated in a variety of human cancers, such as lung cancer, breast cancer, prostate cancer, colon cancer, and liver cancer^22–24^. Some studies showed this lncRNA was involved in the regulation of cell mobility^25^, which was consistent with our findings about cell migration (Fig. 5). H19 has been widely linked to oncogenesis, although the exact mechanism remains unclear^26^. H19 is a precursor of mir-675, which down-regulates tumor suppressor genes in cancer^27^. H19 is also up-regulated in basal cell cancer compared with normal skin specimens^28^, and is associated with poor prognosis in breast cancer^29^. However, it should be note that H19 is down-regulated in high-risk patients in our study, which is inconsistent with these studies. HOTAIRM1 is a kind of lncRNA that plays an important role in the development of immune cells^30^. HOTAIRM1 is also reported to be a tumor suppressor by affecting a series of genes related to cell proliferation in colon cancer^31^.

There were still relatively few studies describing LOC100190986 and AL132709.8 in cancer research. LOC100190986 is associated with HCV genotype 1b transfection in the HepG2 cell line^32^. AL132709.8 was up-regulated in neural precursor cells from patients of lethal congenital contracture syndrome^33^.

The selected six lncRNAs were highly reliable from the perspective of both statistics and biology, since we conducted rigorous internal validation, external validation, and biological explanation. However, our study has some limitations. First, we used different cutoff values for GSE9891 and GSE30161. The overall expression levels of lncRNAs in GSE30161 were lower than that of GSE9891. The main reason for this phenomenon was the different experimental conditions in different labs. We can solve this problem by using a rigorous experimental procedure and adopting a unique cut-off value in the future. Second, the differences of K-M survival curves between low- and high-risk patients were not statistically significant for early-stage patients in GSE9891 (Fig. 4a) and high-grade patients in GSE30161 (Fig. 4f), although patients in the low-risk group still tended to have better DFS. This may be because the sample sizes of these sub-groups were too small (28 cases and 29 cases, respectively). Hence further studies are needed to validate the signature in these groups.

We applied the LASSO method to high-dimensional lncRNA expression data and identified a six-lncRNA signature that was highly associated with OvCa recurrence. This signature was validated in internal and external validation cohorts and was independent of other clinical factors. Furthermore, from a literature review and our functional analysis, we found that the six lncRNAs were closely related with cancer. Thus, this six-lncRNA signature may be a promising method to personalize OvCa therapy and improve patient quality of life according to patients’ condition in the future.

## Methods

### OvCa patient dataset and clinical information

Microarray data for GSE9891 and GSE30161 were downloaded from the Gene Expression Omnibus (GEO; https://www.ncbi.nlm.nih.gov/geo/)^34, 35^. Clinical information for OvCa patients in these data were extracted from the R curatedOvarianData Bioconductor package^36^. The microarray data were measured by the Affymetrix Human Genome U133A Plus 2.0 Array microarray platform. Borderline tumor patients and patients without days to tumor recurrence were excluded from this study. As a result, 257 patients in GSE9891 and 54 patients in GSE30161 were enrolled in this study. The OvCa patients in GSE9891 were randomly divided into a training cohort (n = 100) and an internal validation cohort (n = 157). Additionally, the OvCa patients in GSE30161 were analyzed as an external validation cohort (n = 54).

### Microarray data preprocessing and lncRNA acquisition

The downloaded microarray data for GSE9891 and GSE30161 were normalized using the robust multi-array average (RMA) method^37^. The probe set IDs of lncRNA were acquired from the study of Zhang *et al*.^38^. Briefly, the probe set IDs were mapped to the NetAffx Annotation Files. Based on the Refseq transcripts ID and/or Ensembl gene ID in the annotation files, non-coding RNAs were retained and other types of non-coding RNA except lncRNA were then filtered. Finally, 2446 lncRNAs with corresponding probe set IDs were generated in our study.

### Signature generation and statistical analysis

LASSO penalized regression was conducted to select the lncRNAs associated with OvCa recurrence^39^. The optimal tuning parameter lambda1 was chosen after 100 times of 10-fold cross validation. The function for the selection of lambda1 was “optL1” (fold = 10), and the function for LASSO penalized regression was “penalized” (lambda1 = lambda1). Other parameters of the functions were set to default values. A risk score was generated using the sum of lncRNA expression values weighted by the coefficients from LASSO penalized regression^40^. The OvCa patients were then divided into low- and high-risk groups according to the median risk score.

The association of risk score, clinical factors, disease-free survival (DFS) and overall survival (OS) were assessed by univariate and multivariate cox regression. Kaplan-Meier (K-M) survival curves were used to estimate DFS and OS for patients in the low- and high-risk groups, and the DFS and OS differences between the two groups were assessed using the log-rank test. Time-dependent receiver operating characteristic (ROC) curve analysis with three years as the time point was used to compare the sensitivity and specificity of the DFS prediction based on the risk score^41^. A heat map was used to present the relative expression levels of lncRNAs in this study.

Satterthwaite t-test was performed to determine the significance of each gene, and the corresponding false discovery rate (FDR) value was estimated for correcting multiple comparisons. Differentially expressed genes (DEGs) were selected as FDR < 0.05. Functional annotation of DEGs for Gene Ontology (GO) terms and Kyoto Encyclopedia of Genes and Genomes (KEGG) pathways was performed using the Database for Annotation, Visualization and Integrated Discovery (version 6.7, DAVID, https://david.ncifcrf.gov/) tool^42^. Significant GO biological process terms with similar function were visualized as interaction networks using the Enrichment Map plugin in Cytoscape^43, 44^.

LASSO penalized regression, time-dependent ROC curve analysis and heat map analyses were conducted on penalized^39^, survivalROC^41^ and gplots^45^ packages, respectively, in the R platform. Univariate and multivariate cox regression and log-rank test were performed using SAS (version 9.3, SAS Institute, Cary, NC, USA). K-M survival curves and scatterplots of risk score were performed in GraphPad Prism (version 5.0, Graphpad Software, San Diego, CA, USA)^46^. All other statistical analyses were performed in the R platform (version 3.3.2). All statistical tests were two-sided and a P value of less than 0.05 was considered statistically significant.

## Electronic supplementary material

Supplemental information revision
