# Erratum: Identification of a six-lncRNA signature associated with recurrence of ovarian cancer

**DOI:** 10.1038/s41598-017-07661-3

**Published:** 2017-09-13

**Authors:** Kai Yang, Yan Hou, Ang Li, Zhenzi Li, Wenjie Wang, Hongyu Xie, Zhiwei Rong, Ge Lou, Kang Li

**Affiliations:** 1 Department of Epidemiology and Biostatistics, School of Public Health, Harbin Medical University, Harbin, 150086 P.R. China; 2 Key Laboratory of Cardiovascular Medicine Research, Harbin Medical University, Ministry of Education, Harbin, 150086 P.R. China; 3 Department of Gynecology Oncology, the Tumor Hospital, Harbin Medical University, Harbin, 150086 P.R. China

*Scientific Reports* **7**:752; doi:10.1038/s41598-017-00763-y; Article published online 07 April 2017

The original version of this Article contained an error in the order of corresponding authors.

This has now been corrected in the HTML and PDF versions of this Article.

