# Supplementary material for: Identification of a six-lncRNA signature associated with recurrence of ovarian cancer
Source: Sci Rep. 2017 Apr 7;7:752. doi: 10.1038/s41598-017-00763-y (PMC5429632; doi:10.1038/s41598-017-00763-y)
Supplement: Supplementary file 1 — Supplemental information revision [file 41598_2017_763_MOESM1_ESM.pdf]

Identification of a six-lncRNA signature associated with recurrence of ovarian cancer

Kai Yang[1,†], Yan Hou[1,2,†], Ang Li[1], Zhenzi Li[1], Wenjie Wang[1], Hongyu Xie[1], Zhiwei Rong[1], Ge Lou[3,*] and Kang Li[1,*].

[1] Department of Epidemiology and Biostatistics, School of Public Health, Harbin Medical University, Harbin 150086, P.R. China.

[2] Key Laboratory of Cardiovascular Medicine Research, Harbin Medical University, Ministry of Education, Harbin, 150086, P.R. China

[3] Department of Gynecology Oncology, the Tumor Hospital, Harbin Medical University, Harbin, 150086, P.R. China

[*] Address correspondence to: K.L. (email: likang@ems.hrbmu.edu.cn) or G.L. (email: louge@ems.hrbmu.edu.cn)

[†] These authors contributed equally to this work.

Table S1. Clinical characteristics of patients.

	**GSE9891 training cohort**	**GSE9891 Internal validation cohort**	**GSE30161 External validation cohort**
Number of subjects	100	157	54
Age (median, range)	58(23-80)	60(33-80)	62(38-84)
**Recurrence status**			
No	26	46	6
Yes	74	111	48
**Stage**			
Early[a]	7	21	0
Late[b]	92	136	54
Undocumented	1	0	0
**Grade**			
Low[c]	40	64	21
High[d]	60	91	29
Undocumented	0	2	4
**Histology type**			
Serous	93	146	44
Mucoid	0	0	1
Endometrioid	7	10	1
Clear cell	0	0	5

Undifferentiated	0	0	1
Other	0	1	1
Undocumented	0	0	1

[a]FIGO stage I/II. [b]FIGO stage III/IV. [c]grade 1/2. [d]grade 3.

Table S2. Univariate and multivariate cox regression analyses of OS in GSE9891 and GSE30161.

Variable	Univariate analysis				Multivariate analysis			
	C	P	HR	95% CI of HR	C	P	HR	95% CI of HR
**GSE9891 training cohort (N=100)**								
Risk score	1.7057	**<.0001**	5.505	2.882-10.516	1.6745	**<.0001**	5.336	2.538-11.221
Age	0.0177	0.269	1.018	0.986-1.05	-0.001	0.9577	0.999	0.964-1.035
Stage	16.351	0.9896	12622444	0-.	15.4737	0.9901	5249706	0-.
Grade	0.2268	0.4535	1.255	0.693-2.27	0.4465	0.1845	1.563	0.808-3.023
Histology subtype	15.1456	0.9875	3781311	0-.	15.4452	0.9925	5102479	0-.
**GSE9891 internal validation cohort (N=157)**								
Risk score	1.0028	**0.0058**	2.726	1.337-5.56	0.7026	**0.0708**	2.019	0.942-4.327
Age	0.0317	0.018	1.032	1.005-1.06	0.0266	0.0554	1.027	0.999-1.055
Stage	1.1519	0.0524	3.164	0.988-10.134	0.9403	0.1229	2.561	0.775-8.458
Grade	0.2347	0.3777	1.265	0.751-2.13	-0.1024	0.7111	0.903	0.525-1.552
Histology subtype	1.6525	0.1015	5.22	0.722-37.728	1.0044	0.3349	2.73	0.354-21.032
**GSE9891 entire cohort (N=257)**								
Risk score	1.3261	**<.0001**	3.766	2.305-6.154	1.0828	**<.0001**	2.953	1.777-4.906
Age	0.0248	0.0147	1.025	1.005-1.046	0.0163	0.1255	1.016	0.995-1.038
Stage	1.7359	0.003	5.674	1.8-17.887	1.3809	0.0198	3.979	1.245-12.712

Grade	0.2426	0.2228	1.275	0.863-1.883	0.0081	0.9687	1.008	0.674-1.509
Histology subtype	2.2277	0.0267	9.278	1.294-66.53	1.4232	0.1608	4.15	0.568-30.333
**GSE30161 entire cohort (N=54)**								
Risk score	0.6354	**0.2819**	1.888	0.593-6.006	1.2588	**0.0782**	3.521	0.867-14.296
Age	-0.0035	0.8377	0.996	0.963-1.031	-0.006	0.762	0.994	0.956-1.033
Stage	-	-	-	-	0	.	.	.-.
Grade	0.4046	0.2611	1.499	0.74-3.035	0.5516	0.1421	1.736	0.831-3.626
Histology subtype	0.6695	0.2687	1.953	0.596-6.397	0.5701	0.3742	1.768	0.503-6.218

Abbreviations: C Coefficient, P P value, HR Hazard Ratio, CI Confidence Interval.

Table S3. The detailed GO biological process term information associated with 6 lncRNAs.

Term	Count	%	P-Value	FDR
cell adhesion	193	8.1	5.90E-28	1.10E-24
biological adhesion	193	8.1	7.20E-28	1.40E-24
response to wounding	140	5.9	1.50E-18	2.80E-15
vasculature development	82	3.4	1.00E-16	2.10E-13
blood vessel development	79	3.3	1.20E-15	2.30E-12
inflammatory response	90	3.8	2.70E-13	5.10E-10
immune response	154	6.4	3.40E-13	6.30E-10
extracellular matrix organization	43	1.8	7.40E-13	1.40E-09
blood vessel morphogenesis	66	2.8	1.60E-12	3.00E-09
regulation of response to external stimulus	55	2.3	1.80E-12	3.30E-09
cell motion	110	4.6	1.20E-10	2.30E-07
regulation of cell motion	58	2.4	2.40E-10	4.50E-07
collagen fibril organization	19	0.8	4.80E-10	9.00E-07
regulation of locomotion	57	2.4	5.80E-10	1.10E-06
regulation of cell migration	52	2.2	9.10E-10	1.70E-06
cell migration	71	3	3.60E-09	6.80E-06
extracellular structure organization	49	2.1	6.90E-09	1.30E-05
leukocyte activation	64	2.7	7.50E-09	1.40E-05
positive regulation of immune system process	63	2.6	9.50E-09	1.80E-05
wound healing	54	2.3	1.10E-08	2.10E-05
defense response	126	5.3	1.70E-08	3.20E-05
cell activation	71	3	2.10E-08	3.90E-05
angiogenesis	45	1.9	2.10E-08	4.00E-05
chemotaxis	47	2	3.20E-08	6.00E-05
taxis	47	2	3.20E-08	6.00E-05
positive regulation of response to stimulus	61	2.6	4.30E-08	8.10E-05
positive regulation of developmental process	68	2.8	6.80E-08	1.30E-04
localization of cell	73	3.1	7.20E-08	1.30E-04
cell motility	73	3.1	7.20E-08	1.30E-04
positive regulation of cell differentiation	59	2.5	8.30E-08	1.60E-04
actin cytoskeleton organization	58	2.4	1.30E-07	2.40E-04
T cell activation	39	1.6	1.30E-07	2.40E-04

lymphocyte activation	53	2.2	1.30E-07	2.50E-04
actin filament bundle formation	14	0.6	1.40E-07	2.60E-04
regulation of cell adhesion	41	1.7	1.60E-07	3.00E-04
regulation of inflammatory response	28	1.2	2.20E-07	4.10E-04
heart development	55	2.3	3.10E-07	5.80E-04
positive regulation of cell activation	35	1.5	3.80E-07	7.20E-04
respiratory tube development	33	1.4	4.60E-07	8.60E-04
actin filament-based process	59	2.5	5.40E-07	1.00E-03
positive regulation of cell motion	32	1.3	5.60E-07	1.10E-03
positive regulation of locomotion	32	1.3	5.60E-07	1.10E-03
regulation of cell activation	47	2	5.80E-07	1.10E-03
cell-cell adhesion	65	2.7	5.80E-07	1.10E-03
lung development	32	1.3	7.20E-07	1.40E-03
skeletal system development	71	3	1.50E-06	2.90E-03
tube development	54	2.3	1.60E-06	3.00E-03
cell-substrate adhesion	31	1.3	1.80E-06	3.50E-03
respiratory system development	33	1.4	1.90E-06	3.50E-03
regulation of cell proliferation	144	6	1.90E-06	3.60E-03
positive regulation of cell migration	29	1.2	2.20E-06	4.20E-03
intracellular signaling cascade	212	8.9	2.60E-06	4.80E-03
skeletal system morphogenesis	33	1.4	4.40E-06	8.40E-03
positive regulation of response to external stimulus	23	1	5.30E-06	1.00E-02
cell-matrix adhesion	28	1.2	7.20E-06	1.40E-02
integrin-mediated signaling pathway	24	1	7.80E-06	1.50E-02
regulation of cytokine production	45	1.9	9.30E-06	1.70E-02
regulation of lymphocyte activation	39	1.6	9.80E-06	1.80E-02
positive regulation of T cell activation	25	1	1.10E-05	2.00E-02
positive regulation of leukocyte activation	31	1.3	1.10E-05	2.00E-02
leukocyte adhesion	14	0.6	1.10E-05	2.10E-02
actin filament organization	24	1	1.30E-05	2.50E-02
positive regulation of lymphocyte activation	29	1.2	1.40E-05	2.60E-02
regulation of protein amino acid phosphorylation	43	1.8	1.50E-05	2.90E-02
response to steroid hormone stimulus	46	1.9	2.00E-05	3.80E-02
response to organic substance	129	5.4	2.10E-05	3.90E-02
positive regulation of signal transduction	63	2.6	2.50E-05	4.70E-02
protein kinase cascade	75	3.1	2.60E-05	4.90E-02

Table S4. The detailed GO cellular component term information associated with 6 lncRNAs.

Term	Count	%	P-Value	FDR
extracellular region part	234	9.8	2.00E-24	2.90E-21
extracellular matrix	115	4.8	2.90E-23	4.30E-20
proteinaceous extracellular matrix	108	4.5	2.40E-22	3.50E-19
extracellular region	387	16.2	1.50E-19	2.20E-16
extracellular matrix part	50	2.1	3.60E-15	5.30E-12
plasma membrane part	392	16.4	1.20E-13	1.80E-10
cell surface	90	3.8	4.90E-11	7.30E-08
extracellular space	144	6	8.60E-10	1.30E-06
integral to plasma membrane	222	9.3	1.40E-09	2.00E-06
basement membrane	32	1.3	2.00E-09	2.90E-06
intrinsic to plasma membrane	225	9.4	2.40E-09	3.60E-06
collagen	20	0.8	5.30E-09	7.80E-06
plasma membrane	577	24.2	7.70E-08	1.10E-04
vesicle	134	5.6	8.50E-08	1.30E-04
cytoplasmic vesicle	128	5.4	2.10E-07	3.10E-04
external side of plasma membrane	47	2	4.20E-07	6.20E-04
membrane-bounded vesicle	112	4.7	2.30E-06	3.40E-03
cell leading edge	39	1.6	2.60E-06	3.90E-03
cytoplasmic membrane-bounded vesicle	108	4.5	4.40E-06	6.50E-03
cell-substrate junction	33	1.4	6.90E-06	1.00E-02
actin cytoskeleton	61	2.6	9.40E-06	1.40E-02
cytoplasmic membrane-bounded vesicle lumen	18	0.8	1.40E-05	2.10E-02
receptor complex	33	1.4	1.50E-05	2.20E-02
cell-substrate adherens junction	31	1.3	1.60E-05	2.40E-02
platelet alpha granule lumen	17	0.7	2.10E-05	3.20E-02
fibrillar collagen	9	0.4	2.20E-05	3.20E-02
vesicle lumen	18	0.8	2.80E-05	4.10E-02

Table S5. The detailed GO molecular function term information associated with 6 lncRNAs.

Term	Count	%	P-Value	FDR
glycosaminoglycan binding	48	2	6.80E-11	1.10E-07
growth factor binding	40	1.7	9.20E-11	1.50E-07
extracellular matrix structural constituent	35	1.5	2.10E-10	3.40E-07
polysaccharide binding	50	2.1	2.30E-10	3.70E-07
pattern binding	50	2.1	2.30E-10	3.70E-07
carbohydrate binding	86	3.6	1.20E-09	2.00E-06
actin binding	77	3.2	3.50E-08	5.80E-05
integrin binding	25	1	4.90E-08	8.10E-05
protein complex binding	53	2.2	7.00E-08	1.10E-04
heparin binding	34	1.4	1.60E-07	2.70E-04
platelet-derived growth factor binding	10	0.4	3.20E-07	5.20E-04
cytoskeletal protein binding	100	4.2	2.30E-06	3.80E-03
calcium ion binding	161	6.7	5.00E-06	8.20E-03
extracellular matrix binding	14	0.6	6.40E-06	1.00E-02
GTPase regulator activity	82	3.4	8.80E-06	1.40E-02
nucleoside-triphosphatase regulator activity	83	3.5	1.10E-05	1.90E-02
collagen binding	16	0.7	1.10E-05	1.90E-02
enzyme activator activity	69	2.9	3.00E-05	5.00E-02

Table S6. The detailed KEGG pathway information associated with 6 lncRNAs.

Term	Count	%	P-Value	FDR
ECM-receptor interaction	39	1.6	5.90E-13	7.30E-10
Focal adhesion	65	2.7	3.20E-12	4.00E-09
Cell adhesion molecules (CAMs)	40	1.7	6.10E-07	7.50E-04
Leukocyte transendothelial migration	36	1.5	2.10E-06	2.60E-03
Cytokine-cytokine receptor interaction	63	2.6	2.20E-06	2.70E-03
Chemokine signaling pathway	46	1.9	3.60E-05	4.50E-02

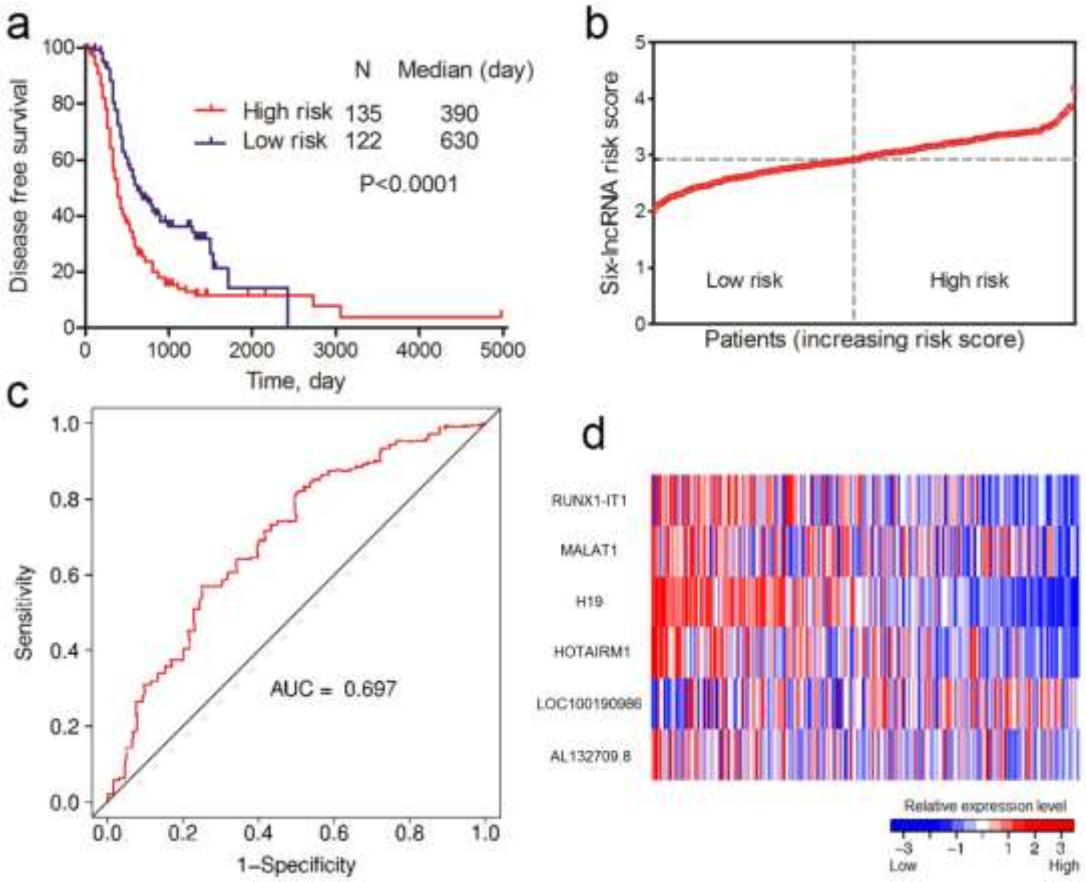

Figure S1. Association between six-lncRNA signature and DFS of OvCa patients in GSE9891 entire cohort. (a) K-M curve of DFS between low- and high-risk patients. (b) Risk scores of each patient in the GSE9891 internal validation cohort (sorted by risk score). (c) Time-dependent ROC curve analysis of the DFS prediction based on the risk score with three years as the time point. (d) Expression heat map of six lncRNAs in OvCa patients in GSE9891 entire cohort (sorted by risk score).

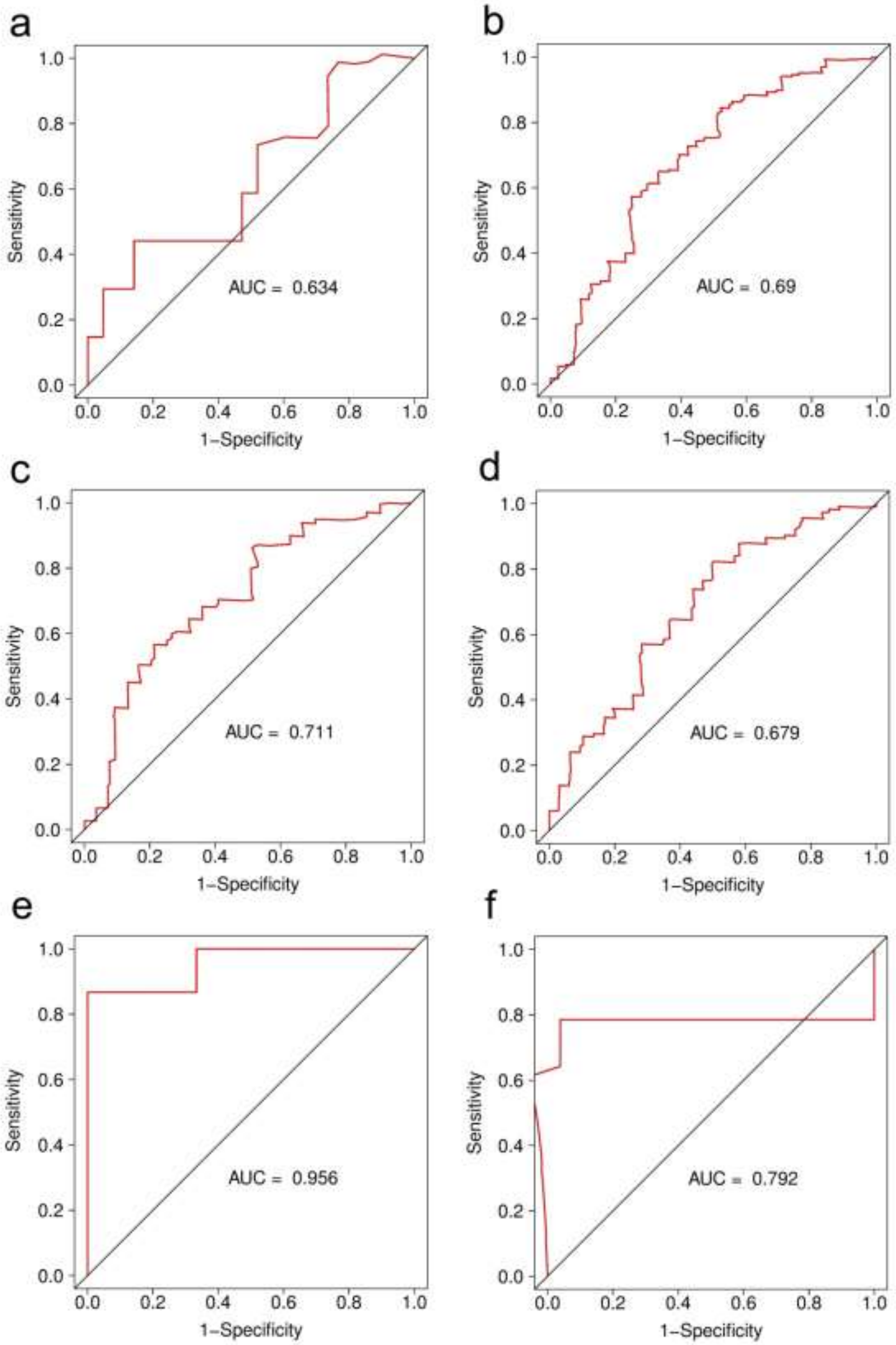

Figure S2. Time-dependent ROC curve analysis of the DFS prediction based on the risk score with 3 years as the time point in sub-groups of OvCa patients. (a)

Early-stage OvCa patients in GSE9891. (b) Late-stage OvCa patients in GSE9891. (c) Low-grade OvCa patients in GSE9891. (d) High-grade OvCa patients in GSE9891. (e) Low-grade OvCa patients in GSE30161. (f) High-grade OvCa patients in GSE30161.

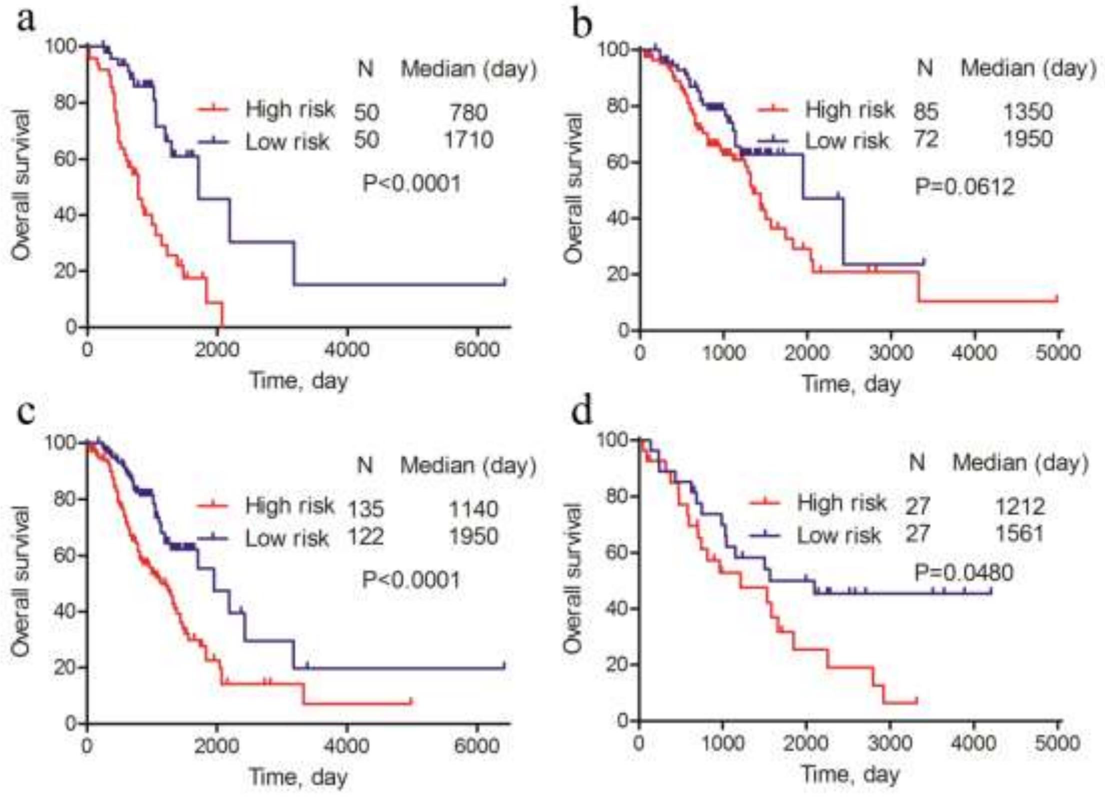

Figure S3. Association between six-lncRNA signature and OS of OvCa patients in different cohorts. (a) K-M curve of OS between low- and high-risk patients in GSE9891 training cohort. (b) K-M curve of OS between low- and high-risk patients in GSE9891 internal validation cohort. (c) K-M curve of OS between low- and high-risk patients in GSE9891 entire cohort. (d) K-M curve of OS between low- and high-risk patients in GSE30161 external validation cohort.
